# Temporal Changes in Ebola Transmission in Sierra Leone and Implications for Control Requirements: a Real-time Modelling Study

**DOI:** 10.1371/currents.outbreaks.406ae55e83ec0b5193e30856b9235ed2

**Published:** 2015-02-10

**Authors:** Anton Camacho, Adam Kucharski, Yvonne Aki-Sawyerr, Mark A. White, Stefan Flasche, Marc Baguelin, Timothy Pollington, Julia R. Carney, Rebecca Glover, Elizabeth Smout, Amanda Tiffany, W. John Edmunds, Sebastian Funk

**Affiliations:** Department of Infectious Disease Epidemiology, London School of Hygiene & Tropical Medicine, London, UK; Department of Infectious Disease Epidemiology, London School of Hygiene & Tropical Medicine, London, UK; Quality Assurance Team, UK-Med Ebola Response Team; IDEA, London, UK; UK Joint Inter-Agency Task Force, International Security Advisory Team Compound, Freetown, Sierra Leone; Health Protection Agency, London, UK; University of Strathclyde, Glasgow, UK; London School of Hygiene & Tropical Medicine, London, UK; Public Health England, London, UK; Department of Infectious Disease Epidemiology, London School of Hygiene & Tropical Medicine, London, UK; Department of Infectious Disease Epidemiology, London School of Hygiene & Tropical Medicine, London, UK; Department of Infectious Disease Epidemiology, London School of Hygiene & Tropical Medicine, London, UK; Department of Infectious Disease Epidemiology, London School of Hygiene & Tropical Medicine, London, UK; Epicentre, Geneva, Switzerland; London School of Hygiene & Tropical Medicine, London, UK; London School of Hygiene & Tropical Medicine, London, UK

**Keywords:** ebola

## Abstract

Background: Between August and November 2014, the incidence of Ebola virus disease (EVD) rose dramatically in several districts of Sierra Leone. As a result, the number of cases exceeded the capacity of Ebola holding and treatment centres. During December, additional beds were introduced, and incidence declined in many areas. We aimed to measure patterns of transmission in different regions, and evaluate whether bed capacity is now sufficient to meet future demand. Methods: We used a mathematical model of EVD infection to estimate how the extent of transmission in the nine worst affected districts of Sierra Leone changed between 10th August 2014 and 18th January 2015. Using the model, we forecast the number of cases that could occur until the end of March 2015, and compared bed requirements with expected future capacity. Results: We found that the reproduction number, R, defined as the average number of secondary cases generated by a typical infectious individual, declined between August and December in all districts. We estimated that R was near the crucial control threshold value of 1 in December. We further estimated that bed capacity has lagged behind demand between August and December for most districts, but as a consequence of the decline in transmission, control measures caught up with the epidemic in early 2015. Conclusions: EVD incidence has exhibited substantial temporal and geographical variation in Sierra Leone, but our results suggest that the epidemic may have now peaked in Sierra Leone, and that current bed capacity appears to be sufficient to keep the epidemic under-control in most districts.

## Introduction

The devastating epidemic of Ebola virus disease (EVD) in West Africa has taken an enormous toll in terms of human suffering and economic loss. As of 18th January 2015, Sierra Leone is the worst affected country, with over 8000 confirmed and probable cases reported^1^. The national and international effort to control EVD in Sierra Leone stepped up considerably between August and December, and a number of new Ebola Treatment Centres (ETCs) have been opened. These units operate high levels of infection control and are used to isolate and provide clinical care to confirmed EVD patients. In addition, Ebola Holding Centres (EHCs) have been constructed as a first destination to assess the status of persons suspected of having EVD and isolate them until confirmatory blood testing, and a number of Community Care Centres (CCCs) - smaller, more lightly staffed - have been opened to help increase bed capacity and bring care closer to communities. This enormous investment in infrastructure has coincided with efforts to improve community engagement and to reduce the risk of transmission, particularly during funerals^2^.

In this study we used a mathematical model of Ebola virus transmission to estimate how the reproduction number, *R*, defined as the average number of secondary cases generated by a typical infectious host, varied between August 2014 and January 2015 in the nine districts of Sierra Leone with the most active transmission. As a large number of additional ETC and EHC beds were introduced in Sierra Leone in December 2014^3^, we also used the model to estimate how many cases would be present in the community by the end of March 2015, and evaluate whether beds currently in place will be sufficient to meet demand.

## Methods

We used a combination of patient and situation report data to obtain reliable and up-to-date time series of the number of confirmed and probable cases. The WHO publishes the weekly number of confirmed and probable cases at the subnational level for Sierra Leone on their website^4^. These data come from the patient database, which is the most reliable data source because it is continuously cleaned. In particular, it takes into account reclassification and avoids double counting of cases. However, the patient database is updated with substantial delay so that the number of reported cases is typically underestimated in the most recent weeks. To tackle this issue, we compiled case data from the daily situation reports issued by the Sierra Leone Ministry of Health and Sanitation (MoHS SitReps) between 10th August 2014 and 18th January 2015^1^. The WHO patient database was used except for the most recent weeks, where we switched to the MoHS SitReps. The time at which this switch was made was determined for each district by comparing the weekly totals from the WHO/patient database and the MoHS SitReps in the 6 weeks preceding publication date of WHO data (11th January 2015). The earliest week where the number of confirmed and probable cases in the MoHS SitReps exceeded that in the WHO/patient database determines this switch. This was between 1 and 4 weeks before. Available bed capacity was compiled for all ETCs, EHCs and CCCs in Sierra Leone^3^.

To model disease transmission, we used a stochastic SEIR transmission model^5^ in which individuals progressed through four classes: susceptible, exposed, infectious and removed. To account for external influences on transmission - such as variation in human behaviour and introduction of control measures - we assumed that the transmission rate could change over time^5^^,^ 6 ^,^ 7 ^,^ 8; the extent and direction of change was estimated during the model fitting process^9^. We used published empirical estimates^10^ of 9.4 days for the mean incubation period and 11.2 days for the mean infectious period (see Appendix for more details).

We fitted the model to the time series of weekly reported cases (confirmed and probable) using a Bayesian approach^9^. As it was not possible to estimate the extent of under-reporting, we fixed the proportion of symptomatic cases reported at 60%, based on recent estimates from the UN for Ebola Emergency Response and the National Emergency Response Centre^2^. We also included the possibility of variability in the accuracy of reporting over time, as well as an over-dispersed delay of 4.3 days in average between onset of symptoms and notification of reported cases^10^. Using the fitted model, we estimated how the reproduction number, *R*, varied between 10th August 2014 and 18th January 2015.

We then used the model to simulate 5000 potential epidemic trajectories from 18th January 2015 until 29 March 2015, and thus to predict the number of cases there would be in the community. Each simulation started with a value of the reproduction number sampled from the posterior distribution on the latest data point. We also conducted a sensitivity analysis by taking the averaged posterior distribution of *R* over the first three weeks of January, in order to smooth over the most recent changes (see Appendix). Upon notification, EVD suspected cases are first sent to an EHC or CCC, where they remain until the result of the laboratory test. Accordingly, we assumed that the number of beds required for EHCs and CCCs at any time was equal to the number of cases in their first three days post-notification (i.e. the average time to obtain the status result), while accounting for the number of non-EVD cases that are also isolated until their laboratory test result (this proportion was computed for each week and district using the reported number of non-EVD cases^1^, see Appendix for more details). Upon confirmation, EVD cases are transferred to ETUs where they may die or recover, so the number of beds required in ETUs was assumed to be equal to the number of cases confirmed but not yet removed. This corresponds to a mean ETC hospitalisation time of 3.9 days^10^ (i.e. the average infectious period minus the average time from onset to laboratory test result).

While this paper was under review, we collected data for two more weeks (weeks ending 25th January and 2nd February 2015). Here, instead of re-fitting to the latest data we decided to assess how well our forecasts matched these two additional data points. Weekly updates of our fit and forecast to the latest data is available online^19^.

## Results

We focused our analysis on the nine districts of Sierra Leone that have reported the most cases since 1st November 2014: Bo, Bombali, Kambia, Koinadugu, Kono, Moyamba, Port Loko, Tonkolili and Western Area (Figure 1 and 2). They have a combined population of 4.7 million, representing 75% of the total Sierra Leonean population. Data source comparison for each district shows that the WHO data published on 11th January 2015 gives the more complete estimate of the number of confirmed and probable cases up to a cut-off date ranging from 30th November 2014 to 4th January 2015, depending on the district (see Figure 2, red lines). After this cut-off date, the situation reports provide more complete estimates of the number of confirmed and probable cases (Figure 2).

**Geographical distribution of Ebola virus disease in Sierra Leone.**
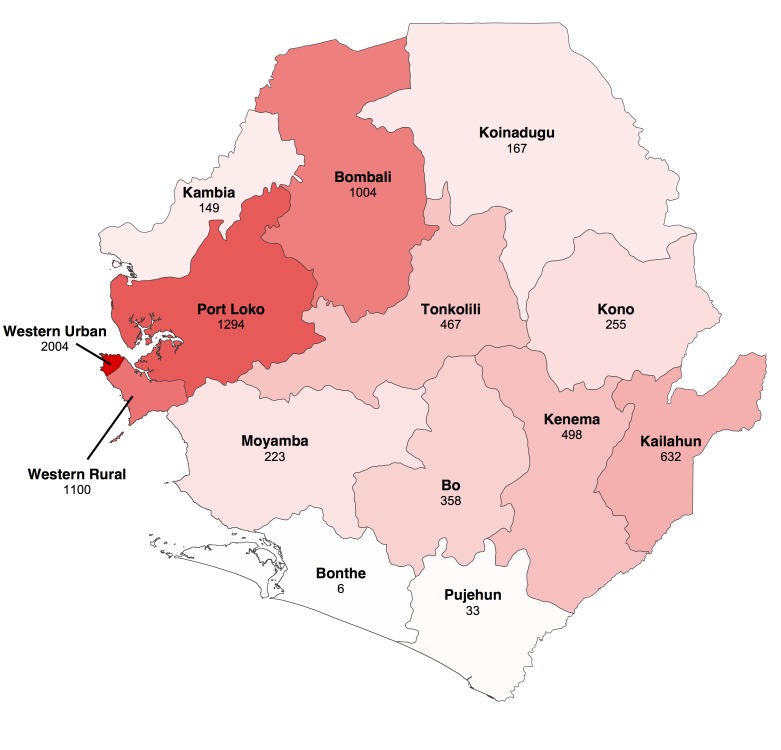
The map shows cumulative number of confirmed and probable cases reported up to 18 January 2015 in the fourteen districts of Sierra Leone. Darker shades of red indicate a greater number of cases.

**Time-series of the weekly number of confirmed and probable cases reported in the patient (black) and situation report (blue) databases.**
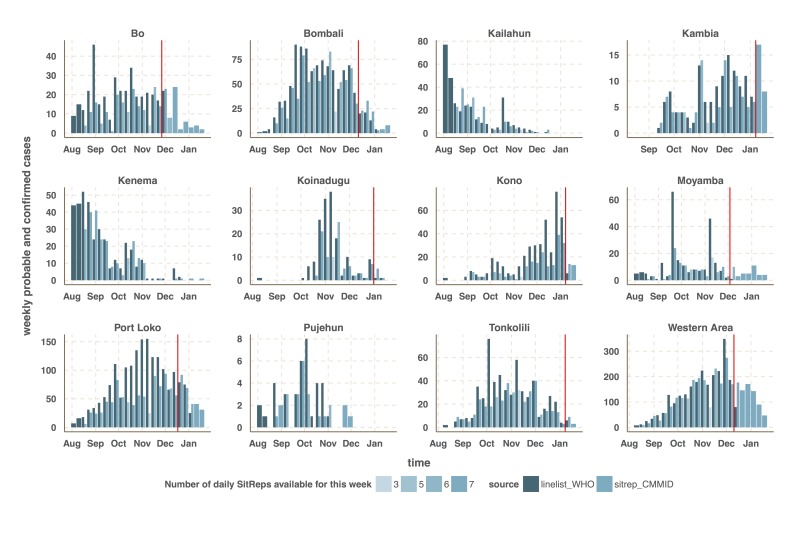
The vertical red line indicates the cut-off date after which we used the situation report database since it provided more reliable information than the patient database.

The estimated number of weekly reported cases in the fitted model was consistent with the observed data, suggesting our framework was able to capture the overall pattern of transmission over time (Figures 3, 4 and 5, upper panels). The epidemic appears to be peaking or in decline in all districts. However, in the most heavily affected districts (Western Area, Port Loko) there were still more than 30 cases per week in January 2015 (Figure 3). The situation is less clear in Kambia, where the number of cases has been stable in January, around 10 cases per week, but only one case was reported on the week ending 2nd February 2015 (Figure 4).

The reproduction number, *R*, generally decreased between August and December in all nine districts (Figures 3, 4 and 5, lower panels). In Western Area, which has had the most cases, we found that the median *R* decreased from 2.8 (interquartile range credible interval, IQR: 2.1-3.8) to 0.32 (0.20-0.47) between August and January and dropped below the critical epidemic control threshold of *R* = 1 in early December (Figure 2). Similar changes occurred earlier in Bo, Bombali, Port Loko and Tonkolili and our median estimate for *R* over January was also below one (Table 1). Accordingly, our forecasts suggest cases could continue to decline in these areas. This trend was confirmed by the two additional weeks of data, except in Port Loko where the number of cases increased on the week ending 2nd February 2015.

**Table 1. Median and interquartile range interval of the posterior estimates of the reproduction number on 18th January 2015 in different districts of Sierra Leone.** 

District	*R (median and IQR)*
Bo	0.35 (0.21 - 0.55)
Bombali	0.28 (0.16 - 0.52)
Kambia	0.97 (0.71 - 1.2)
Koinadugu	0.098 (0.024 - 0.36)
Kono	0.24 (0.078 - 0.63)
Moyamba	0.39 (0.11 - 1.1)
Port Loko	0.46 (0.34 - 0.62)
Tonkolili	0.28 (0.15 - 0.49)
Western Area	0.32 (0.2 - 0.47)

**Model fits and forecast for Bombali, Port Loko, and Western Area.**
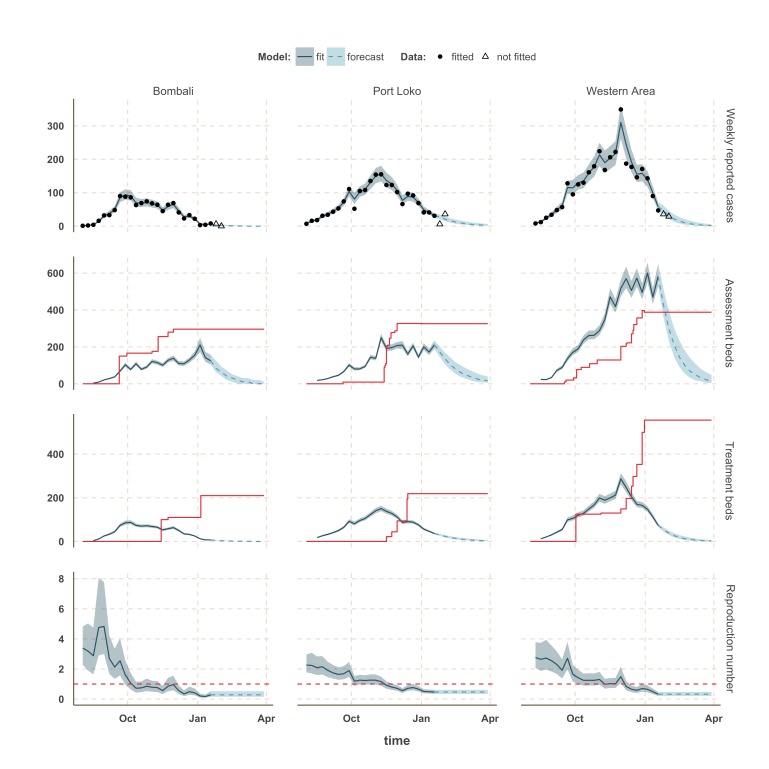
The shaded area is the interquartile range on estimates (grey) and projections (blue), the red solid line is the bed capacity and the red dotted line on the lower panels represents *R*=1, which is the threshold for control. Fitted data are plotted as filled circles and the two additional, non-fitted, data as open triangles.

In Kambia, *R* decreased between September and mid-December from 1.3 (1.1 - 1.8) to 1.0 (0.79 - 1.25) but has since stabilised at around the critical epidemic control threshold (Figure 4). Accordingly, our forecast suggested that the number of cases could either increase, decrease or remain stable over the next few weeks following our latest fitted data point (18th January 2015). Comparing with the two additional weeks of data, we noted that the number of cases remained stable on the week ending 25th January 2015, but dropped below the lower bound of the interquartile range interval (IQR) of our forecast on the following week. Similar drops in the number of cases was also observed in Port Loko and Kono on the first additional week but was followed by an increase on the following week, more in lines with our forecast estimates.

Finally, the situation in Kono, Moyamba and Koinadugu, where *R* has been oscillating around the control threshold since October (Figure 4 and 5), suggest that, although the median *R* were below one on 18th January 2015 and the associated forecasts show a decline of the epidemic, resurgence of cases cannot be ruled out in these areas. In particular, the upper bound of the IQR of *R* in Moyamba is just above one, hence the high variability in our forecast for this district.

**Model fits and forecast for Bo, Kambia, and Kono.**
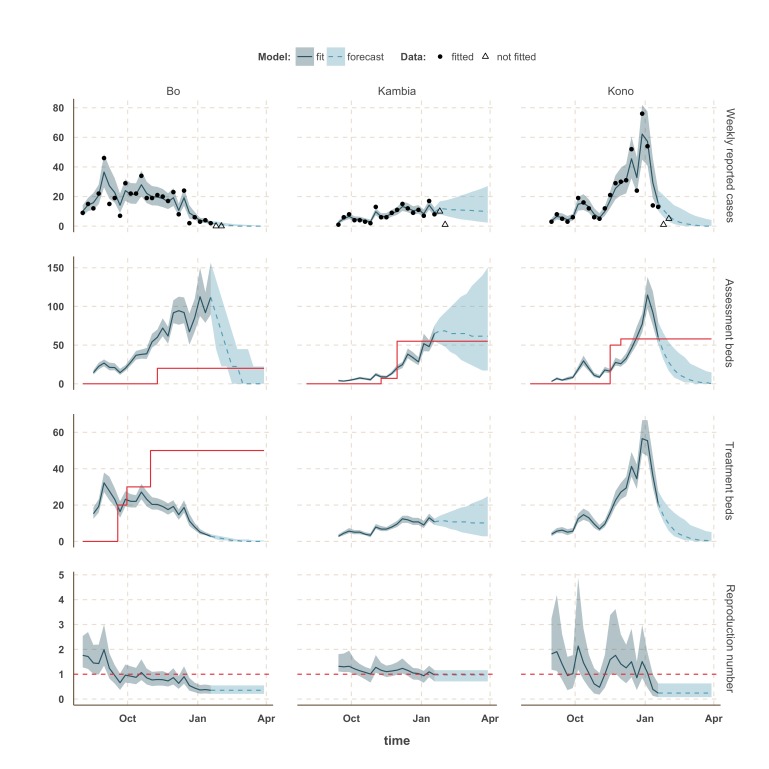
The shaded area is the interquartile range on estimates (grey) and projections (blue), the red solid line is the bed capacity and the red dotted line on the lower panels represents *R*=1, which is the threshold for control. Fitted data are plotted as filled circles and the two additional, non-fitted, data as open triangles.

**Model fits and forecast for Koinadugu, Moyamba and Tonkolili.**
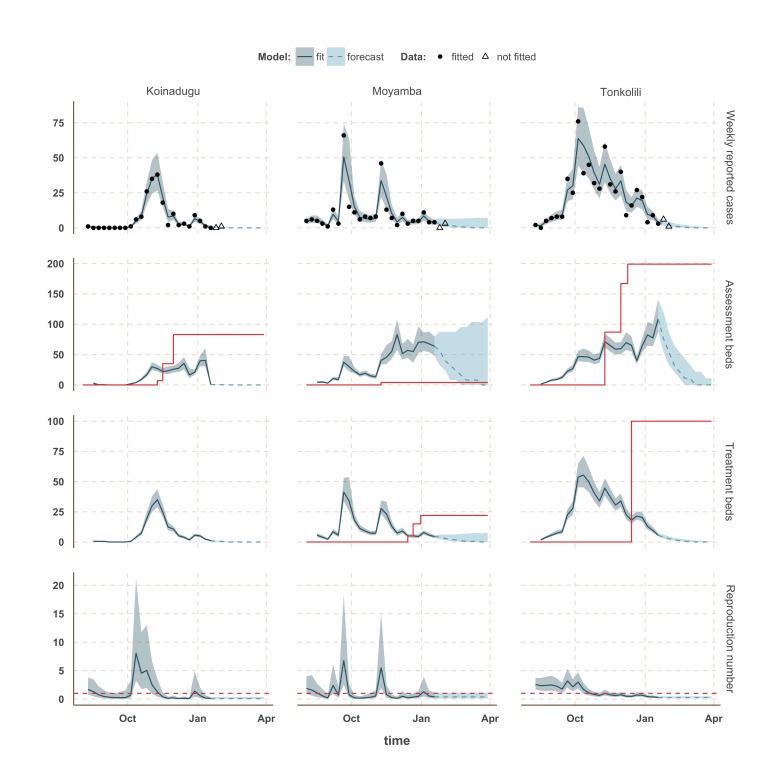
The shaded area is the interquartile range on estimates (grey) and projections (blue), the red solid line is the bed capacity and the red dotted line on the lower panels represents *R*=1, which is the threshold for control. Fitted data are plotted as filled circles and the two additional, non-fitted, data as open triangles.

We used our fitted model to estimate the number of assessment and treatment beds needed over time and compared this with the number of beds available (Table 2). Our results suggest that bed capacity has remained below what was needed since the outset of the Ebola outbreak in most areas, but that this is now changing. In Western Area and Port Loko, for instance, the bed capacity increased dramatically in December, which coincides with the peaking of the epidemic curve (Figure 3). In Bombali and Tonkolili, where the epidemic first started to decline, current bed capacity is predicted to be sufficient (Figure 3 and 5). However, three districts still suffer from a lack of treatment beds (Kambia, Koinadugu, Kono), in particular Kambia, where the assessment bed capacity will become insufficient to isolate all suspected cases in case the epidemic would increase in the near future (Figure 4).

**Table 2. Estimated bed requirements over time in different districts of Sierra Leone (median and IQR):** 

District	Actual assessment beds expected	Assessment beds needed (18th Jan 2015)	Assessment beds needed (29th March 2015)	Actual treatment beds expected	Treatment beds needed (18th Jan 2015)	Treatment beds needed (29th March 2015)
Bo	20	112 (89 - 156)	0 (0 - 22)	50	2 (2 - 3)	0 (0 - 0)
Bombali	296	124 (104 - 156)	0 (0 - 10)	210	6 (5 - 8)	0 (0 - 0)
Kambia	55	64 (54 - 75)	61 (17 - 150)	0	10 (9 - 12)	10 (2 - 24)
Koinadugu	83	0 (0 - 1)	0 (0 - 0)	0	1 (0 - 1)	0 (0 - 0)
Kono	58	60 (50 - 79)	0 (0 - 14)	0	20 (17 - 27)	0 (0 - 5)
Moyamba	4	63 (47 - 79)	0 (0 - 111)	22	4 (3 - 5)	0 (0 - 7)
Port Loko	326	211 (191 - 234)	16 (6 - 39)	219	36 (32 - 40)	2 (1 - 6)
Tonkolili	199	108 (87 - 141)	0 (0 - 10)	100	5 (4 - 7)	0 (0 - 0)
Western Area	388	583 (529 - 651)	13 (4 - 49)	556	73 (66 - 81)	1 (0 - 6)

## Discussion

We compiled data from daily situation reports from Sierra Leone and fitted an EVD transmission model to these reports to estimate how the reproduction number changed in different parts of the country from August 2014 to January 2015. Our analysis suggests that the epidemic is peaking in Sierra Leone, particularly in the more heavily populated Western Area, and that the reproduction number is currently close to or below the epidemic control threshold of *R*=1 in all districts of Sierra Leone. The decline in the reproduction number during December, combined with the ongoing increase in bed capacity, suggest Ebola care facilities have caught up with bed demand in most districts for the first time since the beginning of the outbreak. However, the current situation in Kambia indicates that the number of cases might still increase in the near future. In addition, the rapid changes of *R* around the epidemic control threshold in Kono, Moyamba and Koinadugu since October suggest that resurgence of cases might still occur. This is of concern as these areas are currently under-served by treatment and/or assessment beds. Although opening new ETCs in those areas may not be possible in the coming weeks, rapid opening of new EHCs/CCCs and transfer of confirmed cases to ETUs in neighbouring districts could be envisaged.

We separated the bed demand for EHCs/CCCs from that for ETCs in the model. This is because EHC/CCC planning must anticipate a high proportion of suspected but non-EVD cases. By contrast, we have assumed that ETCs received only confirmed cases. In reality, this separation is subtler as many ETCs proceed to triage and can therefore fill the gap between EHC/CCC capacity and bed demand, such as in Bo.

Our forecast approach assumes that the situation remains unchanged from what is inferred from the last data-point. Comparing our forecasts with two additional weeks of data, we found that this assumption held for the districts showing a steady decline in the number of cases (Bo, Bombali, Koinadugu, Moyamba, Tonkolili and Western Area). In the three other districts the number of cases dropped below our IQR forecast estimates during either the first (Kono and Port Loko) or second (Kambia) additional week. However, the increase in the number of cases during the following week in Kono and Port Loko suggest that one should be cautious in interpreting the recent decline of case in Kambia. By fitting these two additional data points, the model would be able to suggest whether a change in the transmission and/or in the reporting of cases occurred recently in these districts. Finally, we conducted a sensitivity analysis on our forecast by using the average *R* over the last three fitted weeks instead of the last one. We obtained similar results except for Kambia, Kono and Moyamba, where the model forecasted higher number of cases. This is because *R* has been above the control threshold in these districts during the last three fitted weeks.

In many areas the drop in the reproduction number has coincided with an increase in bed capacity. For instance, in Western Area the fall in the reproduction number in October occurred at the same time as several ETCs were opened, notably the Hastings-Freetown ETC organised at the Police Training School (125 beds). However, since we did not include an explicit mechanism by which bed capacity affected transmission in the model^12^^,^ 13 ^,^ 14 we could not measure the extent to which the decline in the reproduction number resulted from more treatment and holding centres versus other factors, such as changes in community behaviour^15^ and burial practice. Indeed, the expansion of bed capacity is likely to partly reflect a general increase in awareness and control efforts. However, such factors are far more difficult to measure than beds. When estimating epidemiological parameters, and effectiveness of interventions, the complexity of a disease model is constrained by the quality of available data^16^. If more data were to become available from Sierra Leone, particularly on the extent of under-reporting and health-seeking behaviour, it would be possible to use a more detailed framework, and thereby examine a potential causal relationship between bed capacity and reduction in transmission.

Community transmission was represented using a single parameter in the model because it has been shown that it is not possible to robustly estimate multiple routes of transmission - such as the contribution from funerals - for Ebola from a single incidence curve^7^. However, knowledge of such factors is not necessary to calculate the change in overall reproduction number over time, and hence understand the average trend in population transmission patterns in real-time^8^^,^ 17. We assumed that the time from onset to hospitalisation and the proportion of reported cases remained constant over time. However, a two-week operation to uncover hidden EVD cases in Western Area occurred at the end of December 2014 and could have lead to a reduction in the time to hospitalisation and to an increase in the proportion of reported cases^18^. We anticipate that this would reduce our estimate of the reproduction number but increase bed requirements, because cases would stay longer in isolation.

Real-time modelling is key to tracking changes in *R* and helping to inform bed capacity planning in the context of the rapidly changing EVD outbreak in West Africa. We are publishing weekly updates of our real-time analysis online^19^.

## Figures and Tables

**Sensitivity analysis on sampling of reproduction number:**
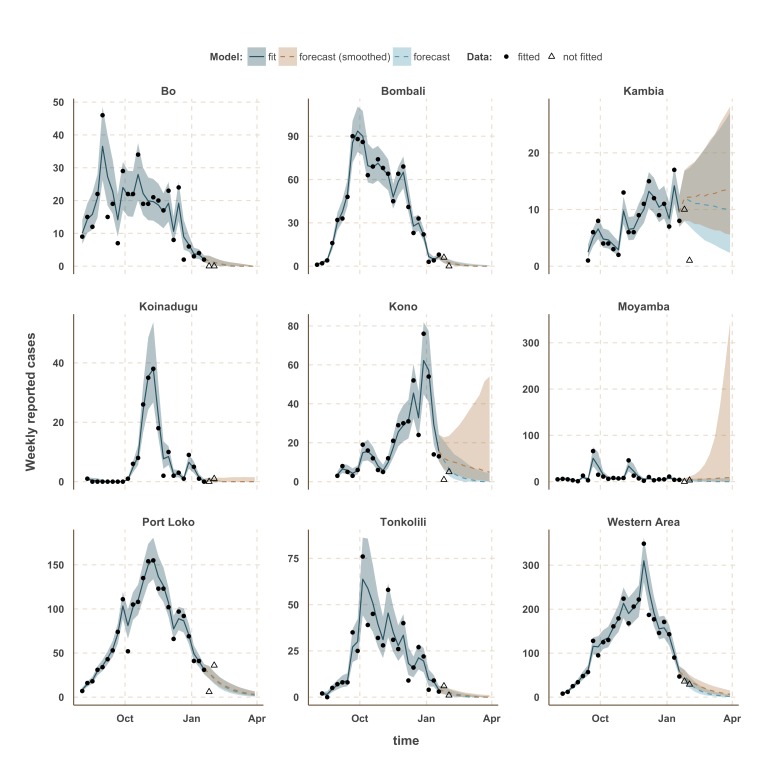
Blue dashed line shows median forecast as in Figures 3-5, with the reproduction number sampled from the posterior at the last data point.Orange dashed line shows median forecast when the reproduction number is sampled from the average posterior distribution over the first three weeks of January. Shaded regions represent interquartile range. Fitted data are plotted as filled circles and the two additional, non-fitted, data as open triangles.

## Appendix 1

### Data

Subnational time-series from the patient database were downloaded from the WHO website^4^ and did not require any post-processing. On the other hand, daily situation reports (SitReps) are generated by the Sierra Leone Ministry of Health and Sanitation (MoHS) and are available on the MoHS website (http://health.gov.sl/?page_id=583). We extracted the data automatically where possible and otherwise manually. In order to smooth day-specific reporting biases, we computed weekly number of new cases by summing daily numbers of confirmed and probable cases. Based on this figure, we selected the nine districts of Sierra Leone with active transmission over the period December 2014 - January 2015: Bo, Bombali, Kambia, Koinadugu, Kono, Moyamba, Port Loko, Tonkolili and Western Area. We grouped the two neighbouring Western Area districts (Rural plus Urban) because patients from one district are commonly sent to ETUs in the other district, depending on bed availability. These two districts form therefore a single area for bed capacity planning. Bed capacity is detailed here http://dx.doi.org/10.6084/m9.figshare.1304889.

### Model and parameters

**Model structure**

To model Ebola virus disease (EVD) transmission, we used a stochastic SEIR framework accounting for hospitalisation and delay in case reporting (Figure 1 and Table 2 of the Appendix). We assumed that the population in each district was initially fully susceptible to infection. Based on published empirical estimates from the WHO Ebola response team^10^, we assumed the incubation period was distributed according to an Erlang distribution with a shape of 2 and a mean, 1/ν = 9.4 days. The infectious period was split between the time commonly spent in the community (I_c_) and hospital (I_h_) by assuming a mean time from onset to hospitalisation, 1/τ = 4.3 days^10^. In our model, we did not explicitly account for status outcome (death/recovery) but grouped all infectious into a single compartment by reweighing the average time from hospitalization to hospital discharge (11.6 days^10^ ) and the average time from hospitalization to death (5.2 days^10^ ) by the case fatality ratio (CFR = 73%^10^ ) in order to obtain an average hospitalization time, 1/γ = 0.73 ∗ 5.2 + (1 − 0.73) ∗ 11.6 = 6.9 days and an average infectious period of 4.3+6.9=11.2 days. This separation of the infectious period between community and hospital allowed us to account for time delay in reporting and to compute the bed demand rather than modelling the mechanistic effect of hospitalisation on the transmission rate, for which we used a phenomenological approach that we describe now.

**Table 2. Description of the transition rates:**

Transition	Description	Rate	Note
* S → E_1_*	Infection	*β_t_S(I_c_ + I_h_)/N*	log(*β*_t_) is a Wiener process^9^. *N* is the population size.
*E_1_ → E_2_*	Progression of incubation	*2vE_1_*	
*E_2_ → I_c_*	Onset of symptoms and infectiousness	*2νE_2_*	
* I_c_ → I_h_*	Hospitalisation and notification	*τI_c_*	Includes multiplicative Gamma noise
* I_h_ → R*	Removal	*γI_h_*	

**Time varying transmission**

We modelled the time-varying transmission parameter, *β*_t_, by a Wiener process^9^, also known as standard Brownian motion, in the log-space (to ensure positivity of *β*_t_):

d log(*β*_t_)=*σ* d*B*_*t*_,

where σ is the volatility of the Brownian motion and was estimated when fitting the model to data. Intuitively, the higher the volatility the larger are the changes in *β_t_* over a given period of time. We used this single parameter to represent transmission as we only had overall incidence data available to fit the model to, rather than detailed case data, hence a more complex model would not have been identifiable^7^.

**The reproduction number**

****The time-varying reproduction number is: *R_t_=β_t_ΔS_t_/N *where Δ is the overall infectious period: Δ=1/τ+1/γ, and *S_t_* is the number of susceptible individuals at time *t*. In all the scenarios studied here, *S_t_*≈*N*. We therefore choose to ignore depletion of susceptibles in our interpretation of *R_t_*≈*β_t_Δ* as the time-varying reproduction number.

**Reporting of cases**

It has been reported that the time from onset to notification of EVD cases is over-dispersed^10^, with a mean of 4.5 days and a standard deviation (s.d.) of 5 days. A similar over-dispersed distribution was reported for the time from onset to hospitalisation (mean 4.6 days and s.d. 5.1 days), which suggests that most notifications occur upon hospitalisation. We modelled this by using a stochastic rate^20^ for the transition leading to hospitalisation of infectious cases (*I*_c_ → *I*_h_). More precisely, we used a multiplicative Gamma noise^1120^ with an infinitesimal standard deviation of 0.1 (to account for the ≈ 10% over-dispersion).

The second source of variability in the weekly incidence data compiled from the SitReps or collected by WHO comes from under reporting. This can be split further between:

- The proportion, *ρ*, of EVD cases that actually report illness. We assumed this proportion was 60% based on reports from the UN for Ebola Emergency Response^2^. We also allowed this parameter to vary over time using an additional over-dispersion parameter, *φ*, that was inferred during model fitting.
- The proportion, *κ_t_*, of daily SitReps that are available each week on the MoHS website and were digitalized. Figure 2 indicates incomplete weeks. For instance, only three SitReps are available for the week 3 to 9 November. Hence the drop in the number of reported cases for that week. We note however that, in this study, we obtained all the daily SitReps (κ_t_=1) for the time period where we use the SitReps instead of the patient database (see Figure 2 of the main text).

Based on these observations, we modelled the weekly incidence reported in the SitReps, *X*_t_, given the simulated incidence *Z*_t_, by a negative binomial distribution with *E*(*X_t_*|*Z_t_*)*=ρκ_t_Z_t_* and *Var*(*X_t_*|*Z_t_*)*=φρ* ^2^ *κ_t_* ^2^ *Z_t_* ^2^.

### Model fitting and inference

For each of the nine districts, we fitted our model and estimated three parameters: over-dispersion of reported cases; volatility (i.e. standard deviation) of the Wiener process on log(*β_t_*); and number of symptomatic cases at the start of the fitting period. We assumed uniform priors for all parameters.

Our inference framework, described below, also allowed us to estimate how the time dependent reproduction number, *R_t_ = β_t_Δ*, defined as the average number of secondary cases generated by a typical infectious host at time t, changed between 10th August and 18th January 2015.

Model fitting was performed using the SSM library^11^ (freely available at: https://github.com/sballesteros/ssm). This library implements a particle Marginal Metropolis-Hastings MCMC algorithm (pMCMC) to perform Bayesian inference for this class of non-linear stochastic models with intractable likelihood^21^.

In SSM, parameters are transformed to ensure positivity (log transform) or any boundedness (e.g. logit transform for probabilities) and the pMCMC is implemented with an adaptive multivariate normal proposal distribution on the transformed parameter space. The adaptive procedure of the proposal kernel operates in two steps. First, the size of the covariance matrix is adapted at each iteration to achieve an optimal acceptance rate of ~23%^22^. Second, its shape is adapted by using the empirical covariance matrix, computed from the accepted samples and updated at each iteration, thus leading to an optimal proposal distribution^23^.

The SSM library also implements a Kalman-simplex algorithm (ksimplex), which was used to maximise the (non-normalized) posterior distribution and thus initialise the pMCMC close to the mode of the target. Since the simplex algorithm only guaranties convergence to a local maximum, we ran 1000 independent ksimplex initialised from parameter sets sampled from the prior distribution. We selected the simplex that converged to the highest posterior density value, and used the outputted parameter set to initialise 2 independent pMCMC chains of 100,000 iterations. We visually checked that the 2 chains converged to the same stationary distribution and combined them after appropriate thinning and accounting for burn-in.  For each posterior sample, a filtered trajectory was sampled from all particles (with probability equal to its overall likelihood). Figures 3, 4 and 5 of the main text show the median and interquartile range of these trajectories at each time point. Figures were plotted using the R software^24^.

### Forecasting and bed requirements

To estimate future bed requirements, we simulated 5000 stochastic trajectories from 18th January 2015 until 29 March 2015. In order to propagate the uncertainty of the Bayesian posterior distribution, each simulation was started by sampling a set of parameters and states from the joint posterior distribution on 18th January 2015 (last fitted data point).

In order to estimate bed requirements for EHCs/CCCs, we accounted for the number of suspected but non-EVD cases who remain isolated until status result. These numbers are reported in the MoHS SitReps and allowed us to compute the weekly proportion of positive EVD cases. Figure 2 of the Appendix shows how this proportion changed over time in each district. Overall, the proportion of EVD cases decreased over time in most districts and was around 30% in January. In the model, we assumed that the bed demand in EHCs/CCCs was equal to the number of EVD cases in their first three days post-notification divided by the empirical proportion of EVD cases at that time. We used the average value over January during the forecast.

In this study, we have used the estimate of the case fatality rate (CFR) for all cases, based on status outcome, which was reported to be 73.4%^10^  in Sierra Leone for the period Dec 2013-25 Nov 2014. Actually, the CFR is lower for hospitalized cases (60.3%) and higher for non-hospitalized cases (91.9%^10^ ).In our model parameterization, decreasing the CFR would lead to an increase in the infectious period, which would have two effects on the inference:

-  Lower estimates of the contact rate (β) in order to obtain the same estimates of the reproduction number over time as when using a shorter infectious period.
-  An increase of the time spent in hospital and thus of the number of beds required.

Accordingly, although a lower CFR would not affect our conclusions regarding the temporal changes in the reproduction number, it would affect our estimates of the number of beds required. For instance, using the CFR of hospitalized cases (60.3% instead of 73.4%) for the whole population would translate into an increase of 22% of the time spent in hospital and thus of the number of beds required. Note however that in our study we assumed that all cases (including the 40% under-reported) would present to the hospital when calculating the number of beds required. As such, our estimates can already be seen as conservative, even without the additional 22% due to a lower CFR in hospitalized patients.

### Sensitivity analysis on model forecast

In the main analysis, we sampled from the posterior distribution of the reproduction number on the latest data point. To conduct a sensitivity analysis, we also took the averaged posterior distribution over the first three weeks of January, which smoothed the most recent changes in reproduction number. Overall, the forecasts remained much the same, except for Kono and Moyamba, where using the average let to a noticeably larger projection of future case numbers, as well as increased uncertainty, which comes from the sudden changes in epidemic dynamics in early January.
